# Influence of Calcium Resonance-Tuned Low-Frequency Magnetic Fields on *Daphnia magna*

**DOI:** 10.3390/ijms232415727

**Published:** 2022-12-11

**Authors:** Viacheslav V. Krylov, Galina A. Papchenkova, Irina L. Golovanova

**Affiliations:** Papanin Institute for Biology of Inland Waters, Russian Academy of Sciences, 152742 Borok, Russia

**Keywords:** daphnia, reproduction, low-frequency magnetic field, static magnetic field, calcium-dependent enzymatic activity, amylolytic activity, sucrase activity, proteolytic activity, Lednev’s model

## Abstract

A biophysical model for calculating the effective parameters of low-frequency magnetic fields was developed by Lednev based on summarized empirical data. According to this model, calcium ions as enzyme cofactors can be the primary target of low-frequency magnetic fields with different parameters tuned to calcium resonance. However, the effects of calcium-resonant combinations of static and alternating magnetic fields that correspond to Lednev’s model and differ by order in frequency and intensity were not studied. It does not allow for confidently discussing the primary targets of low-frequency magnetic fields in terms of the magnetic influence on ions-enzyme cofactors. To clarify this issue, we examined the response of freshwater crustaceans *Daphnia magna* to the impact of combinations of magnetic fields targeted to calcium ions in enzymes according to Lednev’s model that differ in order of magnitude. Life-history traits and biochemical parameters were evaluated. Exposure of daphnids to both combinations of magnetic fields led to a long-term delay of the first brood release, an increase in the brood size, a decrease in the number of broods, and the period between broods. The amylolytic activity, proteolytic activity, and sucrase activity significantly decreased in whole-body homogenates of crustaceans in response to both combinations of magnetic fields. The similarity in the sets of revealed effects assumes that different magnetic fields tuned to calcium ions in biomolecules can affect the same primary molecular target. The results suggest that the low-frequency magnetic fields with parameters corresponding to Lednev’s model of interaction between biological molecules and ions can remain effective with a significant decrease in the static magnetic background.

## 1. Introduction

Electromagnetic load on the environment has constantly increased in recent decades. Anthropogenic sources produce electromagnetic fields in a wide range of frequencies and intensities, among which weak low-frequency magnetic fields are of interest [1,2]. Their energy is too low to overcome the thermal noise threshold [3]. However, significant biological effects of weak low-frequency magnetic fields (changes in the activity of enzymes, reactive oxygen species levels, gene transcription, secretion of hormones, immune cell behavior, neurite outgrowth, cell proliferation, DNA damage, etc.) have been reported many times (see reviews [4,5,6]). Moreover, these effects are resonance-like, i.e., they are repeatedly reproduced at specific frequencies and intensities [7,8,9]. Thus, the regeneration rate in decapitated planarians increased under a magnetic field of 74 μT, 30 Hz and decreased when exposed to a 74 μT, 16 Hz magnetic field [9].

Since the 1980s, several hypotheses that describe putative mechanisms of the influence of weak low-frequency magnetic fields on organisms have been proposed [10,11,12,13,14,15]. Nevertheless, none of them have irrefutable evidence yet [16,17,18]. However, a biophysical model for calculating the effective parameters of low-frequency magnetic fields was developed based on summarized empirical data [19]. According to this model, biological effects will arise under the exposure of an organism to an alternating magnetic field parallel to the static magnetic field (the geomagnetic field in most cases). The effective frequency of the alternating magnetic field formally corresponds to the cyclotron frequency for biochemically important charged particles. An active discussion accompanied the determination of the effective intensity of alternating fields. On the one hand, experiments performed by Blackman’s group measuring the neurite outgrowth rate on PC-12 cells suggested a maximal biological effect caused by the parallel alternating and static magnetic fields with an intensity ratio of approximately Hac/Hdc~0.9 [20]. Combined magnetic fields with this intensity ratio affected calcium efflux from plasma membrane vesicles isolated from *Spinacia oleracea* L. [21] and inhibited growth in cultures of HeLa, VH-10, and Saos-2-His-273 cells [22]. On the other hand, Lednev’s model suggests the maximum biological effects of an alternating magnetic field with an intensity that exceeds that of the static magnetic field by approximately 1.8 times [11]. The effectiveness of this Hac/Hdc ratio has more experimental confirmations that were performed mainly using the organisms but not cell cultures as exposed objects [9,13,23,24,25,26,27,28,29]. Probably, the above-described effects of alternating magnetic fields with different intensities of 0.9 and 1.8 Hac/Hdc regarding Blanchard-Blackman and Lednev’s models [11,12] might be due to the specificity of cells and organisms as objects with different levels of biological organization.

Most of the studies in this field were carried out using the geomagnetic field (within the range of 40–70 µT) as a static component. However, following the above biophysical model, resonant combinations of static and alternating magnetic fields that differ by an order in frequency and intensity can cause the same biological effects [11,19]. The effects of alternating magnetic fields tuned to an ion target and combined with different static magnetic fields were described in a few works evaluating the Blanchard-Blackman model (Hac/Hdc of about 0.9). Thus, Blackman et al. [20] showed that alternating magnetic fields close to resonance peaks of magnesium, vanadium, and manganese ions under a static vertical magnetic field of 36.6 µT inhibit neurite outgrowth in PC-12 cells to the same extent as reduced alternating fields under a static vertical magnetic field of 20.3 µT. Later, the same research group showed similar responses in PC-12 cells to fields tuned to hydrogen ions when studying several alternating magnetic fields with different intensities in combination with static magnetic fields reduced to 2.96 µT and 1.97 µT [30]. However, we could not find papers describing the effects of low-frequency magnetic fields with resonance parameters following Lednev’s model (Hac/Hdc = 1.8) for the same ion under static magnetic fields that differ by orders of magnitude within an experiment with synchronous exposure. To clarify this issue, we performed long-term experiments lasting over two months with freshwater crustaceans *Daphnia magna*.

It should be noted that researchers have repeatedly evaluated the biological effects (changes in planarian regeneration rate, stem cell mitotic activity, and the wound-induced gene expression level; inhibition of opioid peptide-mediated antinociception in land snails) of alternating magnetic fields in a range of frequencies and amplitudes in short-term tests previously [9,13,23]. These studies confirm the maximum effects of combinations of static and alternating magnetic fields corresponding to Lednev’s model. In the present paper, we tested the hypothesis that different magnetic fields tuned to calcium resonance following Lednev’s model can cause the same effects in the biochemical and life-history traits of *D. magna*. We evaluated the influence of two calcium-tuned magnetic fields: on the background of a geomagnetic field (52 µT static MF, 94 µT 40 Hz alternating MF) and an order of magnitude lower geomagnetic field (5.2 µT static MF, 9.4 µT 4 Hz alternating MF). The geomagnetic field and reduced by order of magnitude geomagnetic field were used as reference conditions. This approach allowed us to obtain a sufficient sample size in a limited volume of Helmholtz coils. Life-history traits, general amylolytic activity, general proteolytic activity, and sucrase activity were utilized to evaluate the biological effects of magnetic fields. Previously, it was shown that these characteristics are sensitive to magnetic influences [27,31,32,33].

## 2. Results

**Figure 1:**
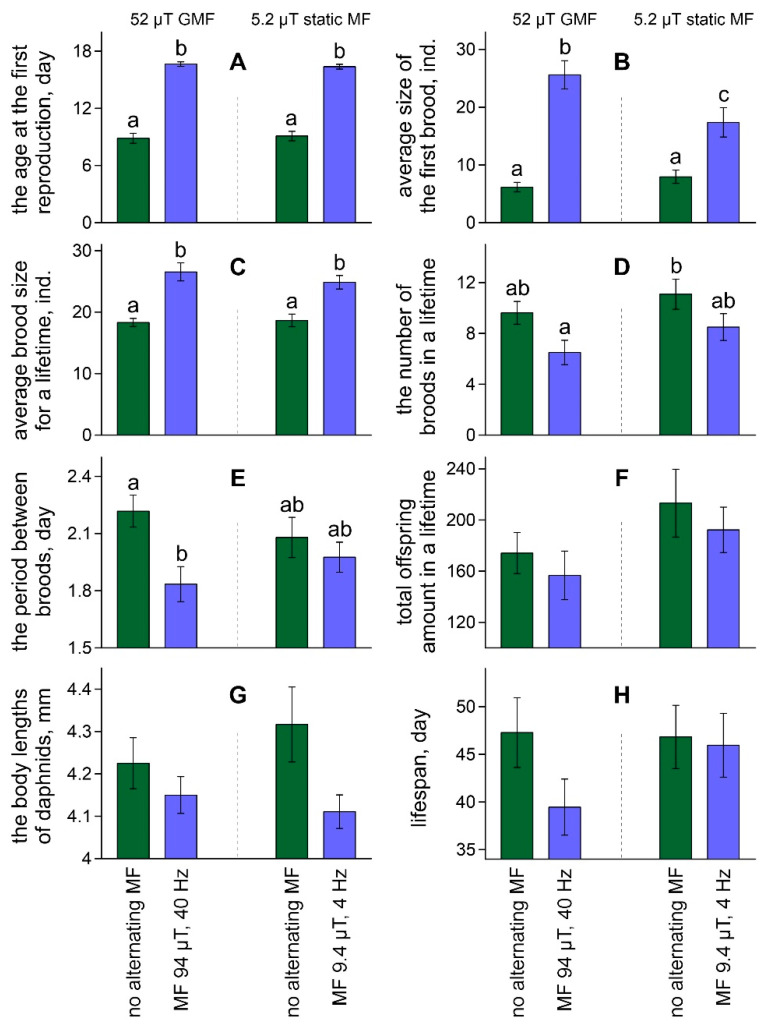
Age at the first reproduction (**A**), size of the first brood (**B**) and averaged brood size for a lifespan (**C**), the number of broods (**D**), the period between broods (**E**), the total offspring amount (**F**), the body lengths of parental daphnids (**G**), and its lifespan (**H**) in the groups of *D. magna* exposed to studied magnetic fields. Values are means ± standard error. Significant differences (*p* < 0.05) among groups after Tukey’s post-hoc multiple comparison tests indicated by different letters.

Both combinations of the alternating magnetic field with resonance parameters for calcium ions significantly affected the age at the first reproduction (F[1,83] = 361.71, ŋ^2^ = 0.81, *p* < 0.001), average brood size in the first brood (F[1,83] = 56.82, ŋ^2^ = 0.41, *p* < 0.001), brood size for a lifetime (F[1,83] = 42.45, ŋ^2^ = 0.34, *p* < 0.001), the number of broods for a lifetime (F[1,83] = 7.63, ŋ^2^ = 0.08, *p* < 0.01), the period between broods (F[1,83] = 7.20, ŋ^2^ = 0.08, *p* < 0.01), and the body lengths of daphnids (F[1,58] = 5.9, ŋ^2^ = 0.09, *p* < 0.05). The average values of these characteristics for each group are shown in Figure 1. The direction and magnitude of the effects caused by both alternating magnetic fields with different parameters were the same. The influence of the static magnetic field intensity and the interaction of factors did not affect or have little impact on the studied characteristics (Tables S1 and S2).

**Figure 2:**
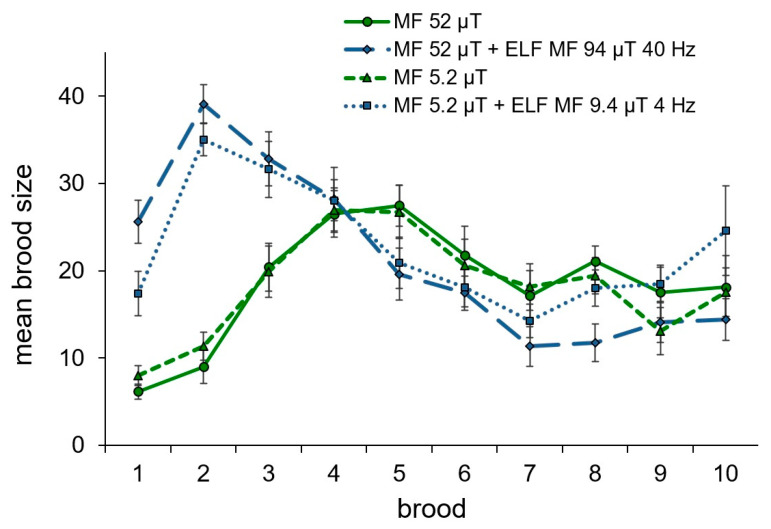
Dynamics of changing mean brood size (ind.) in the in the groups of *D. magna* exposed to studied magnetic fields. Bars denote standard error.

The effect of the alternating magnetic fields with resonance parameters for calcium ions on daphnids’ reproduction consisted of a long-term (for more than a week) significant delay of the first brood release compared with females maintained with no alternating fields (Figure 1A). At the same time, the size of the first brood and the average brood size in daphnids from the groups exposed to the alternating magnetic fields were significantly greater than that in animals maintained without alternating fields (Figure 1B,C). The dynamics of changes in the size of the first ten broods are shown in Figure 2. The total number of broods and the period between broods in females exposed to the alternating magnetic fields were lower than that in animals maintained without alternating fields (Figure 1D,E). However, significant differences were recorded between daphnids maintained in the ten-times reduced magnetic field and alternating magnetic field combined with the geomagnetic field for the total number of broods and between daphnids maintained in the geomagnetic field and alternating magnetic field combined with the geomagnetic field for the period between broods. Statistically insignificant trends towards a reduction in the number of offspring produced, a decrease in body size, and life expectancy under the exposure to the alternating magnetic field with resonance parameters for calcium ions were observed both in the geomagnetic field and in the ten-times reduced magnetic field (Figure 1F–H).

**Figure 3:**
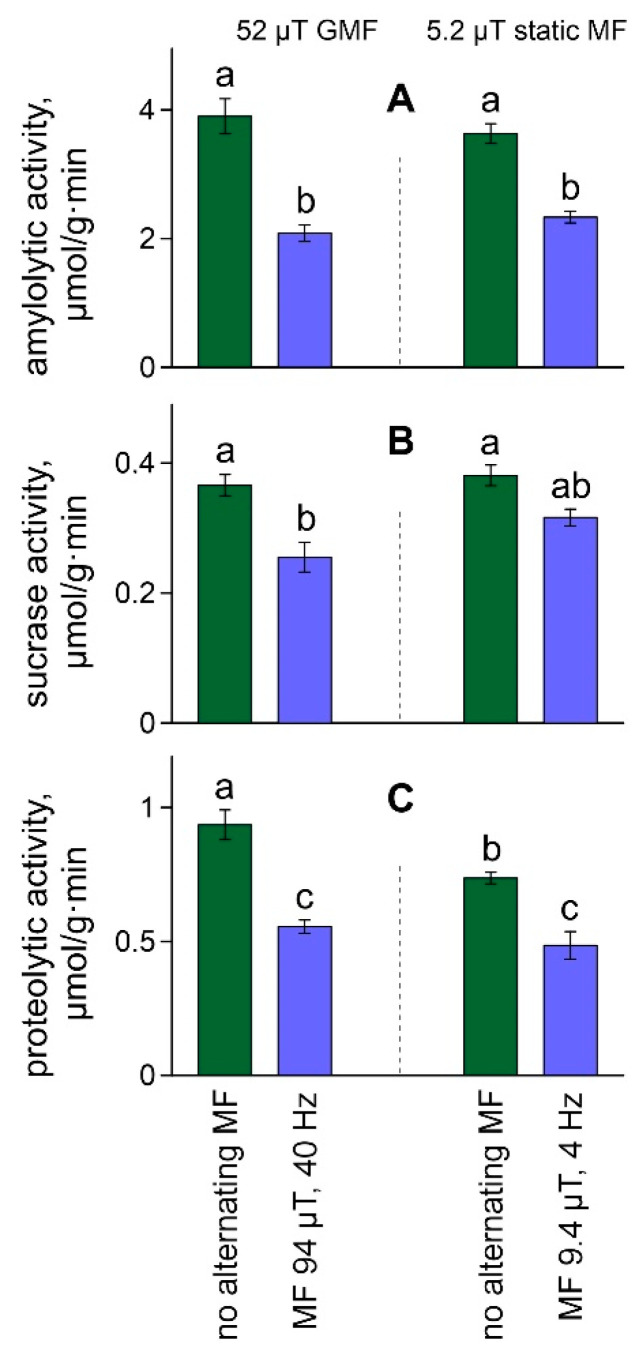
The amylolytic activity (**A**), sucrase activity (**B**), and proteolytic activity (**C**) in the whole-body homogenates of daphnids exposed to studied magnetic fields. Values are means ± standard error. Significant differences (*p* < 0.05) among groups after Tukey’s post-hoc multiple comparison tests indicated by different letters.

The activity of digestive enzymes in whole-body homogenates showed the same pattern. Both magnetic fields with resonance parameters for calcium ions significantly affected amylolytic activity (F[1,16] = 79.48, ŋ^2^ = 0.83, *p* < 0.001), sucrase activity (F[1,16] = 25.19, ŋ^2^ = 0.61, *p* < 0.001) and proteolytic activity (F[1,16] = 58.32, ŋ^2^ = 0.78, *p* < 0.001). In all cases, enzyme activities decreased under the influence of alternating magnetic fields (Figure 3). In addition, the static magnetic field intensity affected proteolytic activity in daphnia to a lesser extent (F[1,16] = 10.58, ŋ^2^ = 0.40, *p* < 0.01) due to a decrease in this parameter with a reduction in the intensity of the static field (Figure 3C). The interaction of factors did not affect the studied characteristics (Table S3).

## 3. Discussion

The magnetic fields with resonance parameters for calcium ions depressed the reproduction of crustaceans. The same effects, including a delay in the first reproduction for 1–2 days, were previously reported for 500 Hz, 150 μT magnetic fields on the background of the geomagnetic field [31], and the reversal of the static magnetic field [34]. It should be noted that the parameters of magnetic exposure in the above studies were not consistent with the current biophysical models [11,15]. Alternating magnetic fields with calcium-resonance parameters led to a longer delay in the first reproduction of daphnids in the present experiment. This result could be associated with the repression of the reproductive system development or the resorption of the eggs in the first broods in response to the calcium-tuned magnetic fields [35]. The first three broods were registered as larger in daphnids exposed to these fields compared to both groups maintained without alternating fields. Sizes of the first broods in *D. magna* exposed to the magnetic fields with resonance parameters for calcium ions approximately correspond to the third-fourth broods produced by daphnids from reference groups. It also suggests the possible resorption of the first few broods in groups exposed to the low-frequency magnetic fields. The numerous first broods led to increased average brood size in the groups exposed to the alternating magnetic fields. However, due to the reduction in the number of broods, the total amount of offspring born in lines exposed to the low-frequency magnetic fields remained lower than that maintained in static fields. These results are consistent with previously published data on the negative impact of low-frequency magnetic fields on the reproductive potential of *D. magna* [31,36].

The low-frequency magnetic fields influence organisms according to general biophysical patterns [19]. The decrease in amylolytic activity, proteolytic activity, and sucrase activity are also consistent with the effects of low-frequency magnetic fields obtained in other species. A decline in the activity of alpha- and beta amylases of the wheat seeds was registered after its exposure to a magnetic field (16 Hz, 5 mT) for 2 h over 13 days [37]. The influence of a magnetic field with resonance parameters for calcium ions (Bdc = 24.2 μT, Bac = 44.5 μT, Fac = 18.5 Hz) caused a decrease in amylolytic activity and proteolytic activity in the intestines of crucian carp *Carassius carassius* (L.) yearlings after an in vivo exposure for 1 h [27]. Exposure of juvenile tilapia (*Oreochromis niloticus* L., 1758) to magnetic fields with 50 Hz frequency and intensity of 30, 100, 150, or 200 μT for 30 days led to a significant decrease in the activity of intestinal protease [38]. A decrease in amylolytic activity and sucrase activity was revealed in roach underyearlings as delayed consequences of magnetic influence during embryogenesis [33,39]. The decline in daphnids’ proteolytic activity in the reduced static magnetic field in the present study is consistent with the data on the reduction in the static magnetic field influence on digestive and intracellular proteinases [27,40] and confirms a non-species-specific biochemical response to low-frequency magnetic fields.

We find the only paper that describes the response of *D. magna* to magnetic fields with resonance parameters for calcium ions [26]. The influence of the combination of static and alternating magnetic fields (Bdc = 24.2 µT, Bac = 44.5 µT, Fac = 18.5 Hz) led to a dramatic decrease in calpain activity in the whole-body homogenates of juvenile crustaceans [26]. Despite the differences between calpains and digestive proteinases, the reduction in enzyme activity in response to the exposure to magnetic fields with resonance parameters for calcium ions was the same as in the present experiment.

Following Lednev’s biophysical model, a primary target in the chain of events that are launched by the low-frequency magnetic fields is a calcium ion bound with the calcium-binding center of a protein that possesses calcium-dependent enzymatic activity. The bound calcium in this model is regarded as an isotropic charged oscillator excited by thermal fluctuations. The potential in which the excited calcium ion moves has spherical symmetry (i.e., the considered oscillator is isotropic). In a static magnetic field, a degenerate vibrational level with a frequency ω will split into sublevels due to the Zeeman effect. The oscillations of magnetic sublevels are coherent. The given coherence provides interference interaction between sublevels of calcium oscillators and a response to the influence of calcium-resonance-tuned low-frequency magnetic fields [11]. These fields affect the affinity of calcium to the calcium-binding center of a protein [19].

The effects of calcium-tuned magnetic fields on α-amylase were expected as it is a calcium-dependent enzyme. The calcium ion is located near the active center of this protein and performs a stabilizing function [41]. Sucrase and serine proteinases do not require calcium, but these ions are involved in biochemical processes that may modulate the functioning and localization of the studied enzymes [42,43]. The effects found in life-history traits could be a consequence of modulations in digestive enzymes. In addition, other calcium-dependent enzymes and processes [43] could contribute to the changes in the life history of exposed daphnids.

The almost complete coincidence of biological effects in response to different alternating magnetic fields with resonance parameters for calcium ions is noticeable in the present study. It is known that the effects of slightly different low-frequency magnetic fields can be radically different [9,36]. Previously several studies revealed the similar effects of alternating magnetic fields tuned to ion targets according to Blanchard-Blackman’s model (Hac/Hdc of about 0.9) under different static magnetic fields [20,30]. In this experiment, low-frequency fields differed significantly in frequency and amplitude but tuned to the same target according to Lednev’s model [19]. The results of the present study are the first evidence that Lednev’s biophysical model (Hac/Hdc = 1.8) is also applicable under static magnetic fields that significantly differ from the geomagnetic field. It provides prospects for magnetic controlling biochemical and other ion-dependent processes via magnetic fields with resonance parameters. The absence of dependence between effects expression and the frequency and intensity of the magnetic fields suggests the quantum nature of magnetic influence on primary targets in cells.

## 4. Materials and Methods

### 4.1. Test Animals

*D. magna* culture was isolated from a seasonal pool (Voronezh region, Russia). It was maintained and used in experiments at the I.D. Papanin Institute for Biology of Inland Waters of the Russian Academy of Sciences for more than twelve years. The sensitivity of *D. magna* to toxicants was within standard norms [44].

Animals were maintained at 22–23 °C in water media prepared by adding soluble components to distilled water according to the American Society for Testing and Materials protocol [45]. The media was changed twice a week. The dissolved oxygen level was measured daily with an oximeter HI 9146 N (Hanna instruments, Romania). It varied in the range of 6.8–7.2 mg/L. The photoperiod was 16 h day/8 h night. Daphnids were fed daily with *Chlorella vulgaris* Beijerinck cell suspension (3–3.5) × 10^5^ cells/mL. Algae were cultured in Bold’s basal medium and concentrated with a bucket-type centrifuge (K23D; MLW, Leipzig, Germany).

### 4.2. Magnetic Fields

Two different combinations of magnetic fields were used in the experiments:

1. A 5.2 µT static magnetic field and a 9.4 µT 4 Hz alternating magnetic field;
2. A 52 µT static magnetic field (the geomagnetic field) and a 94 µT 40 Hz alternating magnetic field.

Both combinations fit predicted effective or resonant conditions for calcium ions regarding Lednev’s model [11,19]. It means that the primary targets of these different magnetic fields should be equal.

The sinusoidal magnetic fields were created in two standard signal generators (G6-27 and G3-56 models, Radiopribor, Velikie Luki, Russia) and transferred to two pairs of Helmholtz coils (0.5 m in diameter, 700 turns of 0.2 mm copper wire in each coil, see Supplementary Figure S1). The coils were placed two meters away from each other and directed parallel (collinear) to the geomagnetic field. The direction of the geomagnetic field was determined with a three-component flux-gate magnetometer NV0302A (ENT, St. Petersburg, Russia). The Helmholtz coils that generated a 9.4 µT 4 Hz alternating magnetic field had additional 700 turns of 0.2 mm copper wire over the main winding. This additional winding was supplied with direct current from a DC supply (AKIP-1103, Manson Engineering Industrial, Hong Kong, China) to provide the static magnetic field opposite the geomagnetic field. Thereby, the resulting static magnetic field within the working volume of Helmholtz coils was reduced to 5.2 µT due to the superposition. The third pair of Helmholtz coils was supplied with direct current from the DC supply but no alternating signal. Parameters of static and alternating magnetic fields were controlled daily using an NV0599C flux-gate magnetometer (ENT, St. Petersburg, Russia).

### 4.3. Structure of the Experiments and Evaluated Parameters

The experiments were carried out in 50 mL polypropylene vials (40 mm diameter, 40 mm height) containing 40 mL of the media. Eighty-eight neonates not older than 24 h were sampled randomly from the culture of *D. magna* and transferred individually into 88 vials using a plastic pipette. The crustaceans were split into four groups of 22 animals. These groups were placed in the following conditions:

1. the geomagnetic field of 52 µT (control);
2. ten-times reduced magnetic field of 5.2 µT;
3. alternating magnetic field of 9.4 µT, 4 Hz combined with the reduced magnetic field of 5.2 µT;
4. alternating magnetic field of 94 µT, 40 Hz combined with the geomagnetic field of 52 µT.

The four groups were exposed to different magnetic conditions simultaneously. Experiments were carried out for 71 days until the natural death of all crustaceans. The number of live females and the number of offspring produced by each female were recorded daily. This procedure takes about 30–40 min for each group. The vials with the daphnids were taken away from exposure systems for this period. Age at the first reproduction, lifespan, mean brood size, the period between broods, and the number of broods for a lifespan were determined from the observed life-history characteristics. The body lengths of parental animals (distance from the head to the base of the caudal spine) were measured after their death. Measurements were performed using an MC-2 microscope (Micromed, Saint-Petersburg, Russia) equipped with a DCM-500 camera (Hangzhou Huaxin IC Technology, Hangzhou, China), using Image-Pro 3.0 software (Media Cybernetics, Bethesda).

In order to determine the activity of enzymes, the offspring of females from the third to sixth broods were collected from each group and snap-frozen in liquid nitrogen immediately after the counting. Since the water medium and daphnia bodies are penetrable to static and low-frequency magnetic fields, the juveniles were exposed to the same influences as the parental individuals. Total enzyme-active homogenates were prepared from several hundred juveniles of each experimental group using cooled (2–4 °C) Ringer’s solution (110 mM NaCl (Shostka Chemical Reagents, Russia), 1.9 mM KCl (Shostka Chemical Reagents, Russia), 1.3 mM CaCl_2_ (Shostka Chemical Reagents, Russia), pH 7.4). The substrate solutions (soluble potato starch 18 g/L (Merck, Millipore Sigma-Aldrich Supelco), sucrose 50 mM (Merck, Millipore Sigma-Aldrich Supelco), and casein 10 g/L (Merck, Millipore Sigma-Aldrich Supelco)) were prepared with the same Ringer’s solution. The enzyme-active homogenates and substrate solutions were mixed and incubated for 20–30 min while constantly stirring. This procedure was performed at 20 °C and pH 7.4.

The amylolytic activity, which reflects the sum of activities of the enzymes hydrolyzing starch (α-amylase EC 3.2.1.1, glucoamylase EC 3.2.1.3, and maltase EC 3.2.1.20), and sucrase activity (EC 3.2.1.48) were evaluated using the modified Nelson’s method [46] through an increase in hexoses. Proteolytic activity (sum activity of serine proteinases, trypsin, EC 3.4.21.4, chymotrypsin, EC 3.4.21.1, and activity of tri- and dipeptidases) was assayed by determining the increase in tyrosine by the method of Anson [47]. Absorbance was determined using a spectrophotometer (Lambda 25, PerkinElmer, Waltham, MA, USA). The activity of enzymes was determined in five replications separated before the homogenization and expressed as the micromoles of the reaction products derived from 1 min of incubations per 1 g wet tissue (μM/g min).

A two-way ANOVA was performed to test for the influence of alternating magnetic fields and changes in static magnetic field intensity on the studied characteristics (four groups of 21–22 observations each). The normality (Shapiro–Wilk W test) and homoscedasticity (Levene’s test) assumptions were satisfied. The ANOVA results were reported with an F-statistic, η2 as a measure of effect size, and *p*-value. Tukey’s post-hoc multiple comparison tests were performed to determine the significance of the differences between groups.

## 5. Conclusions

The present study assumes that magnetic fields tuned to calcium ions according to Lednev’s model and significantly differed in frequency and amplitude may affect the same primary target. It should be noted that Lednev’s model cannot explain all biological effects of ELF MF published to date [18]. A step forward was the development of a general physical mechanism for the biological effects of weak magnetic fields [15], which includes Lednev’s parametric resonance in biosystems. However, further research is needed to define all primary targets for the low-frequency magnetic fields and the transformation pathways from these primary targets to the biological responses at higher levels.

## Data Availability

All data generated or analyzed during this study are included as Supplementary Files “Biochemistry.xlsx” and “Life-history.xlsx”.

## Supplementary Materials

The supporting information can be downloaded at: https://www.mdpi.com/article/10.3390/ijms232415727/s1.
