# Supplementary material for: Influence of Calcium Resonance-Tuned Low-Frequency Magnetic Fields on Daphnia magna
Source: Int J Mol Sci. 2022 Dec 11;23(24):15727. doi: 10.3390/ijms232415727 (PMC9779586; doi:10.3390/ijms232415727)
Supplement: Supplementary file 1 [file ijms-23-15727-s001.zip › supplementary.pdf]

Supplementary information

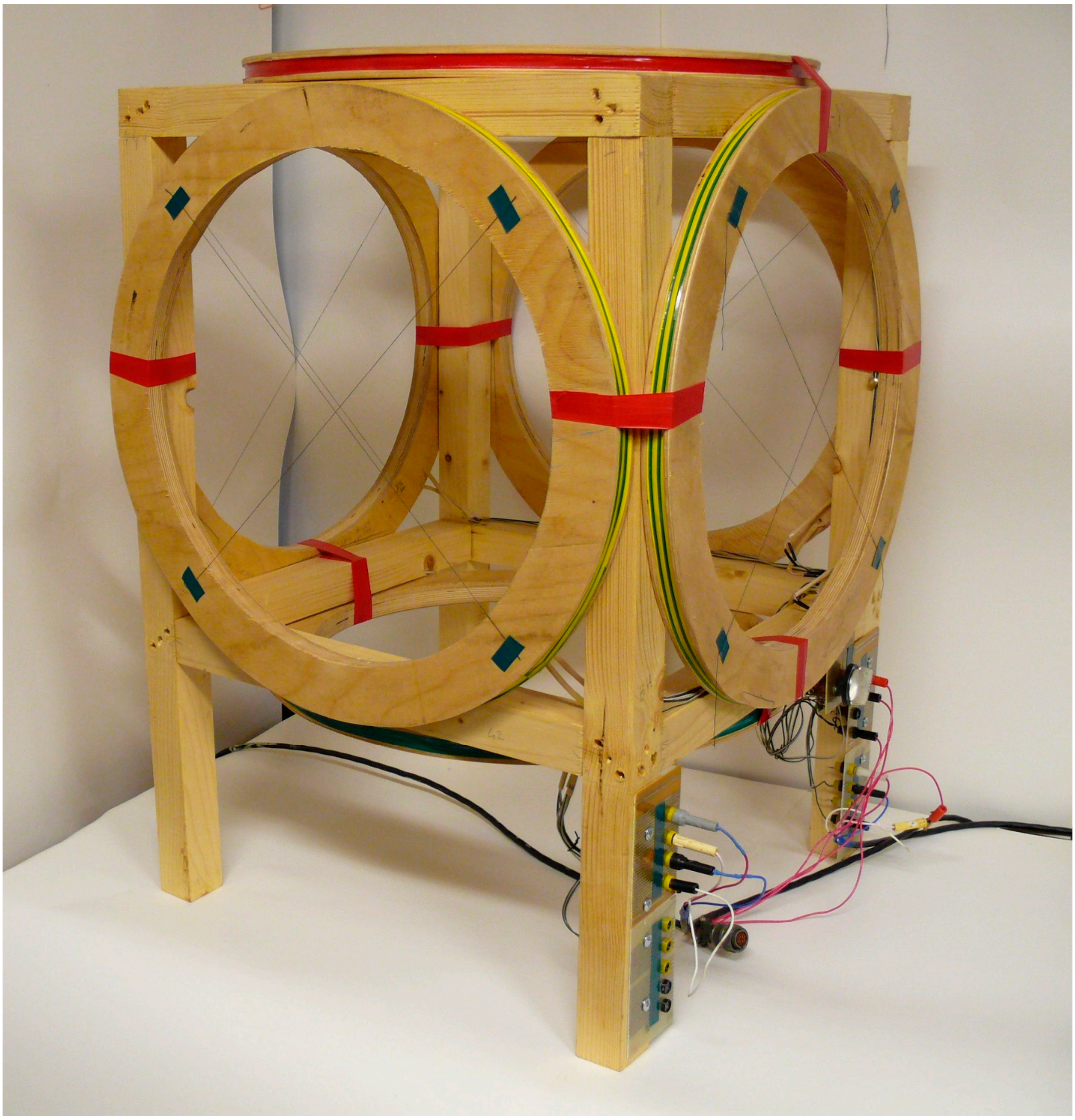

Figure S1. System of coils used for the generation of alternating magnetic fields and reduction of the geomagnetic field. Each coil is 0.5 m in diameter and contains two windings.

Table S1. Two-way ANOVA on life-history traits of daphnids from experimental groups.

	Age at the first reproduction			Brood size in the first brood			Brood size for a lifetime		
Source of variation	d.f.	Mean square	F	d.f.	Mean square	F	d.f.	Mean square	F
[1] Changes in static magnetic field intensity (52 μT or 5.2 μT)	1	0.01	0.002	1	223.72	2.808	1	9.90	0.371
[2] The presence of Ca^{2+}-alternating magnetic field	1	1231.43	361.708***	1	4526.16	56.817***	1	1131.49	42.451***
Interaction [1]x[2]	1	1.39	0.410	1	547.77	6.876*	1	22.20	0.833
Error	83	3.40		83	79.66		83	26.65	

F Fisher test is the variance ratio (mean square between classes/mean square within classes); d.f. degrees of freedom; asterisks indicate statistical significance: *p<0.05; ***p<0.001.

Table S2. Two-way ANOVA on life-history traits of daphnids from experimental groups.

	The number of broods for a lifetime			The period between broods			The body lengths of daphnids		
Source of variation	d.f.	Mean square	F	d.f.	Mean square	F	d.f.	Mean square	F
[1] Changes in static magnetic field intensity (52 μT or 5.2 μT)	1	65.516	2.821	1	0.001	0.001	1	0.010	0.21
[2] The presence of Ca^{2+}-alternating magnetic field	1	177.210	7.630**	1	1.287	7.201**	1	0.295	5.90*
Interaction [1]x[2]	1	1.516	0.065	1	0.426	2.382	1	0.064	1.28
Error	83	23.226		83	0.179		58	0.050	

F Fisher test is the variance ratio (mean square between classes/mean square within classes); d.f. degrees of freedom; asterisks indicate statistical significance: *$p<0.05$; **$p<0.01$.

Table S3. Two-way ANOVA on biochemical characteristics of daphnids from experimental groups.

	Amylolytic activity			Activity of sucrase			Proteolytic activity		
Source of variation	d.f.	Mean square	F	d.f.	Mean square	F	d.f.	Mean square	F
[1] Changes in static magnetic field intensity (52 μT or 5.2 μT)	1	0.001	0.002	1	0.007	4.762*	1	0.090	10.580**
[2] The presence of Ca^{2+}-alternating magnetic field	1	12.142	79.475***	1	0.038	25.190***	1	0.499	58.320***
Interaction [1]x[2]	1	0.334	2.159	1	0.003	1.714	1	0.021	2.420
Error	16	0.153		16	0.002		16	0.009	

F Fisher test is the variance ratio (mean square between classes/mean square within classes); d.f. degrees of freedom; asterisks indicate statistical significance: *p<0.05; **p<0.01; ***p<0.001.
